# A Real-World Analysis of the Safety and Efficacy of Teclistamab for Patients with Relapsed/Refractory Multiple Myeloma and Baseline Renal Impairment—USMIRC Group

**DOI:** 10.3390/cancers18050740

**Published:** 2026-02-25

**Authors:** Maha Hameed, Alma Habib, Abdullah Mohammad Khan, Mehak Masood Laharwal, Prerna Mewawalla, Marshall McKenna, Yun Kyuong Ryu Tiger, Mansi Shah, Hira Shaikh, Christopher Strouse, Kimberly Green, Jordan Snyder, Zahra Mahmoudjafari, Muhammad Umair Mushtaq, Nausheen Ahmed, Al-Ola Abdallah, Shebli Atrash, Barry Paul, Reed Friend

**Affiliations:** 1 Internal Medicine, Florida State University, Sarasota, FL 34239, USA; 2 Division of Hematologic Malignancies & Cellular Therapeutics, University of Kansas Medical Center, Westwood, KS 66205, USA; 3 Division of Hematology, The Ohio State University, Columbus, OH 43210, USA; 4 Division of Hematology and Cellular Therapy, Allegheny Health Network Cancer Institute, Pittsburgh, PA 15224, USA; 5 Rutgers Cancer Institute, New Brunswick, NJ 08901, USA; 6 Division of Hematology, Oncology, and Blood & Marrow Transplantation, University of Iowa, Iowa City, IA 52242, USA; 7 Division of Hematology-Oncology, Medical University of South Carolina, Charleston, SC 29425, USA; 8 Levine Cancer Institute, Atrium Health Wake Forest University School of Medicine, Charlotte, NC 28204, USA

**Keywords:** teclistamab, renal impairment, cytokine release syndrome, efficacy, toxicity

## Abstract

Background: Multiple myeloma (MM) is a hematologic malignancy characterized by the clonal proliferation of plasma cells and a rapidly evolving treatment landscape. Bispecific antibodies targeting B-cell maturation antigens (BCMA) have emerged as promising therapeutic options for relapsed and/or refractory multiple myeloma (RRMM). Methods: This retrospective study evaluated the efficacy and safety of teclistamab, a BCMA-directed bispecific antibody, in patients with RRMM with renal impairment (RI) at baseline compared to those without. Conducted across seven academic centers, the study included 195 patients with RRMM, of whom 34 had baseline RI. Results: Performance status, previous lines of treatment, and prior BCMA exposure were identified as significant predictors of progression-free survival. Notably, patients with RI demonstrated overall response rates and toxicity profiles comparable to those without RI, although they required more packed red blood cell transfusions. No statistically significant differences were observed in adverse events, including cytokine release syndrome and infections. Conclusions: Overall, the findings support the efficacy and safety of teclistamab in patients with RRMM and RI, highlighting the need for prospective clinical trials to optimize the treatment strategies for this population.

## Simple Summary

Teclistamab is a bispecific antibody used for treating relapsed/refractory multiple myeloma. The use of teclistamab in patients without renal impairment has demonstrated well-tolerated outcomes; however, its efficacy and safety in patients with renal impairment have yet to be closely explored. This retrospective study aimed to compare outcomes in patients with relapsed/refractory multiple myeloma with and without renal impairment who received teclistamab.

## 1. Introduction

Multiple myeloma (MM) is a plasma cell disorder that accounts for 1% of all cancers and 10% of all hematologic malignancies [1]. Diagnosis requires the presence of one or more myeloma-defining events, in addition to 10% or more clonal plasma cells on bone marrow biopsy [1]. The standard of care for transplant-eligible newly diagnosed multiple myeloma (NDMM) is 4–6 cycles of quadruplet induction therapy followed by autologous stem cell transplantation (ASCT). In transplant-ineligible NDMM, primary therapy is tailored to the frailty profile, with more fit, younger individuals preferred to receive quadruplet regimens, while those with greater frailty are often treated with triplet combinations. Therapies available for NDMM include a combination of an anti-CD38 monoclonal antibody, immunomodulatory agent, proteasome inhibitor and dexamethasone. Alternative therapies include alkylator-based regimens. In relapsed/refractory multiple myeloma (RRMM), treatment has become increasingly nuanced as the therapeutic landscape rapidly expands. Over the past five years, numerous novel FDA-approved agents, including cellular therapies such as bispecific antibodies and chimeric antigen receptor T-cell therapy (CAR-T), have become more widely available and in turn have significantly improved patient outcomes.

B-cell maturation antigen (BCMA), also known as tumor necrosis factor receptor superfamily member 17 (TNFRSF17), is expressed on mature B cells and plasma cells, as well as malignant plasma cells, and engages with the ligands A Proliferation-Inducing Ligand (APRIL) and B-cell activating factor (BAFF) to cause upregulation of the Nuclear factor kappa-light-chain-enhancer of activated B cells (NF-kB) pathways, c-Jun N-terminal kinase (JNK), Mitogen-activated protein kinase (MAPK), and Phosphoinositide 3-kinase/protein kinase B (PI3K/AKT) pathways, leading to further growth and immunosuppression to promote malignant plasma cell survival and further drive myeloma progression [2,3].

Bispecific antibodies, which work by engaging immune T-cells to kill myeloma cells, can have different target antigens including BCMA. In BCMA bispecific antibodies, the membrane protein promotes plasma cell survival in multiple myeloma and exists as surface proteins and free soluble forms [4]. In 2022, teclistamab-cqyv became the first FDA-approved T-cell redirecting bispecific antibody for use as monotherapy in patients with triple-class-exposed RRMM [4]. Teclistamab is a bispecific antibody that targets the CD3 receptor complex on T-cells and the BCMA receptor on plasma cells of multiple myeloma, thereby facilitating dual binding of both receptors for T-cell activation, cytokine release, and the release of perforin/granzymes to cause myeloma cell death [4]. Importantly, teclistamab has been well tolerated in patients with RRMM when administered using a step-up dosing regimen (0.06 mg/kg and 0.3 mg/kg subcutaneously), followed by a maintenance dose of 1.5 mg/kg weekly, with the option to extend to every two weeks after achieving a response [5]. This regimen was used in the landmark MAJESTIC-1 clinical trial, in which 165 patients who received teclistamab achieved an overall response rate of 63% and a median progression-free survival of 11.3 months. Further, patients had a median duration of response of 18.4 months over a median follow-up of 14.1 months. However, patients with a creatinine clearance (CrCl) ≤ 40 mL/min were excluded from the trial, resulting in limited data for this population. [5,6].

Several other phase I-III clinical trials involving the use of teclistamab in RRMM have been reported in the literature: MajesTEC-2, -3, -4, -5, -7, -9, TriMM-2, -3, and RedirectTT-1 [7,8,9,10,11,12,13], all of which demonstrated favorable efficacy outcomes with the use of teclistamab. Similar to MAJESTEC-1, these trials excluded patients with moderate-to-severe RI. A recent case report described a favorable response in a patient with ESRD and RRMM treated with teclistamab followed by talquetamab and elotuzumab while undergoing peritoneal dialysis [14]. Another report demonstrated the feasibility and safety of elranatamab in a triple-class RRMM with end-stage renal disease on hemodialysis [15]. However, there is a paucity of literature regarding the efficacy of teclistamab in treating RRMM in patients with baseline RI. This retrospective study aimed to investigate the efficacy and safety of teclistamab in patients with RRMM with RI compared to those without baseline RI.

## 2. Methods

This multi-institutional retrospective study was conducted across seven academic centers in the US, including all consecutive patients who received treatment with teclistamab between 8/2022 and 7/2024, in collaboration with the U.S. Myeloma Innovations Research Collaborative (USMIRC). Patients with RRMM, with and without RI, who received teclistamab therapy were included. The 2021 CKD-EPI equation recommended by the National Kidney Foundation (NKF) is a race-free formula used to estimate the Glomerular Filtration Rate (GFR) from serum creatinine, age, and sex [16]. It removes race-based coefficients, promoting health equity and test standardization for all patients. The equation is: eGFR = 142 × min(SCr/κ,1)α × max(SCr/κ,1) − 1.200 × 0.9938^Age^ × 1.012 [if female]. RI was defined as a creatinine clearance of <40 mL/min. Baseline characteristics, including age, sex, race, performance status (PS), response to previous therapy, immunoglobulin type, kappa/lambda ratio, cytogenetics, and prior ASCT history, were collected.

Responses to therapy were evaluated using the IMWG response criteria [17]. Continuous variables were summarized and reported as mean (min, max) and median (IQR), as well as dichotomized factors by total number/frequency. Categorical variables were summarized using frequency counts and percentages. Fisher’s exact test was used to analyze contingency tables, the Wilcoxon rank-sum test was used to compare two independent samples, and Pearson’s chi-squared test was used to compare independent and dependent samples. The Kaplan–Meier method was used to determine progression-free survival (PFS). The false discovery rate correction was used for multiple test analyses. When indexed to patient baseline characteristics, PFS univariate and multivariate analyses were also conducted to report efficacy outcomes using the hazard ratios (HR) and 95% confidence intervals (CI). Statistical significance was set at *p* > 0.05.

We utilized the R Core Team (2024) for descriptive analysis [18]. Safety outcomes, including toxicity adverse events (AEs) of cytokine release syndrome (CRS), infection, cytopenia, leukopenia, anemia, neutropenia, and thrombocytopenia, were assessed at 30-day and 90-day intervals. The use of interventions such as Granulocyte Colony-Stimulating Factor (GCSF), thrombopoietin (TPO) agonists, intravenous immunoglobulin (IVIG), packed red blood cells (pRBC), and platelet transfusions in response to AEs has also been documented. AEs were graded according to the National Cancer Institute Common Terminology Criteria for Adverse Events (CTCAE, version 4.03) [19]. CRS and immune effector cell-associated neurotoxicity syndrome (ICANS) were graded according to the American Society for Transplantation and Cellular Therapy consensus grading system [20].

The study was conducted in accordance with the Good Clinical Practice Guidelines [21], and the study protocol, amendments, and relevant documents were approved by the institutional review board at each study site.

## 3. Results

### 3.1. Baseline Characteristics

Between August 2022 and June 2024, 195 patients with RRMM were treated with teclistamab; 34 (17.4%) had baseline RI. Patients with RI were slightly older (median age 71 vs. 69 years), but there were no significant differences in sex, race, or performance status between groups. The distribution of R-ISS stage and immunoglobulin or light-chain subtype was comparable. High-risk cytogenetic abnormalities, including del(17p), t(4;14), t(14;16), and 1q gain, were similarly represented across cohorts. ASCT and the number of previous lines of treatment did not differ significantly. Exposure and refractoriness to major drug classes (proteasome inhibitors, IMiDs, anti-CD38 antibodies, and BCMA-directed therapies) were also balanced, although a trend toward higher proteasome-inhibitor and IMiD refractoriness was observed in patients with RI. Overall, baseline demographic, disease, and treatment characteristics were well matched between patients with and without RI.

Supplementary Table S1 shows the baseline patient characteristics of those with and without RI. The median follow-up period was 11 months (IQR, 6.00; 14.00).

### 3.2. Efficacy Outcomes

IMiD refractory status (*p* = 0.01; HR = 1.99; 95% CI, 0.73–5.42)), double refractory status (*p* = 0.041; HR = 5.16; 95% CI, 0.68–38.9)), weekly dosage regimen (*p* = 0.022; HR = 4.92; 95% CI, 1.04–23.4), and ORR (*p* = <0.001; HR = 2.12; 95% CI, 0.48–9.30) were significantly associated with shorter PFS on univariate analysis in patients with RI.

Notably, baseline characteristics, including age, sex, race, PS, R-ISS stage, cytogenetics, and previous therapy responses, demonstrated comparable PFS outcomes in multivariate analysis when associated with PFS in patients with RI. However, ORR was statistically significant in both univariate and multivariate analyses of patients with RI when associated with PFS.

Univariate analysis of the entire patient cohort revealed that age (*p* = 0.026; HR = 0.98; 95% CI, 0.96–1.00), performance status (*p* = 0.002), presence of 1q gain mutation (*p* = 0.04; HR = 1.64; 95% CI, 1.12–2.41), number of treatment lines prior to teclistamab (*p* = 0.014), carfilzomib refractory status prior to teclistamab use (*p* = 0.03; HR = 1.73, 95% CI, 1.02–2.95), lenalidomide refractory status prior to teclistamab (*p* = 0.005; HR = 2.16; 95% CI, 1.2–3.86), daratumumab exposure prior to teclistamab use (*p* = 0.031; HR = 0.13, 95% CI, 0.03–0.52), BCMA exposure prior to teclistamab use (*p* = 0.023; HR = 1.56; 95% CI, 1.06–2.27), and presence of extramedullary disease (*p* = 0.002; HR = 1.88; 95% CI, 1.28–2.76) were statistically significant outcomes when indexed to PFS. Supplementary Table S2 demonstrates the impact of each baseline patient characteristic on PFS in the univariate analysis of RRMM patients with RI treated with teclistamab.

**Figure 1:**
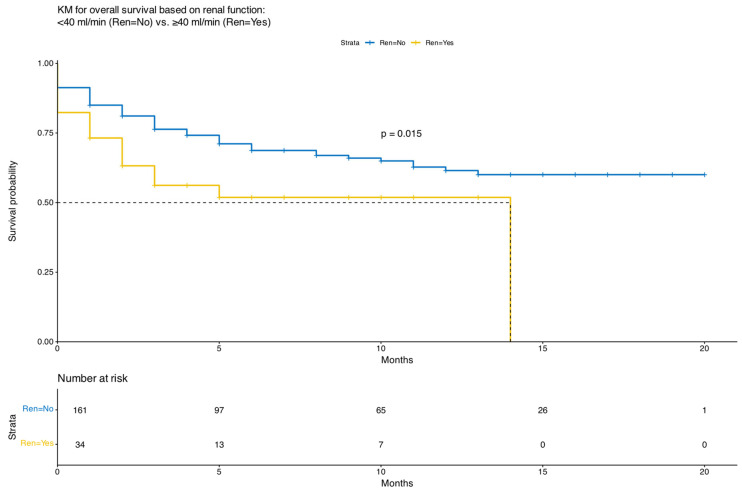
Kaplan–Meier Curve for Overall Survival of Teclistamab in Patients with Renal Impairment.

**Figure 2:**
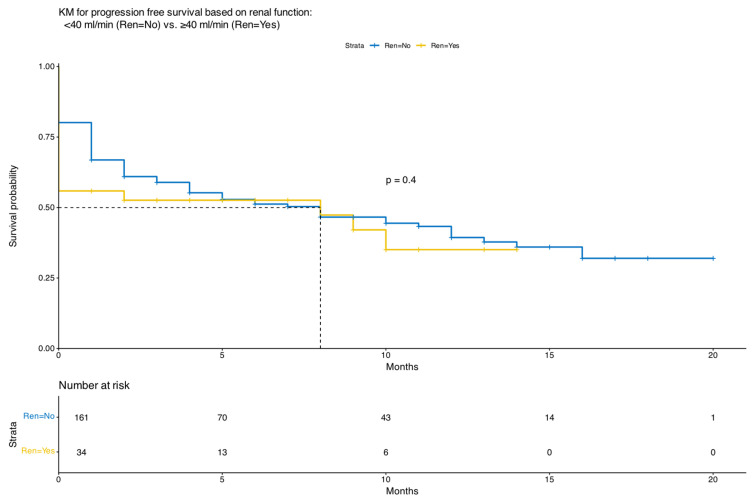
Kaplan–Meier Curve for Progression Free Survival of Teclistamab in Patients with Renal Impairment.

**Figure 3:**
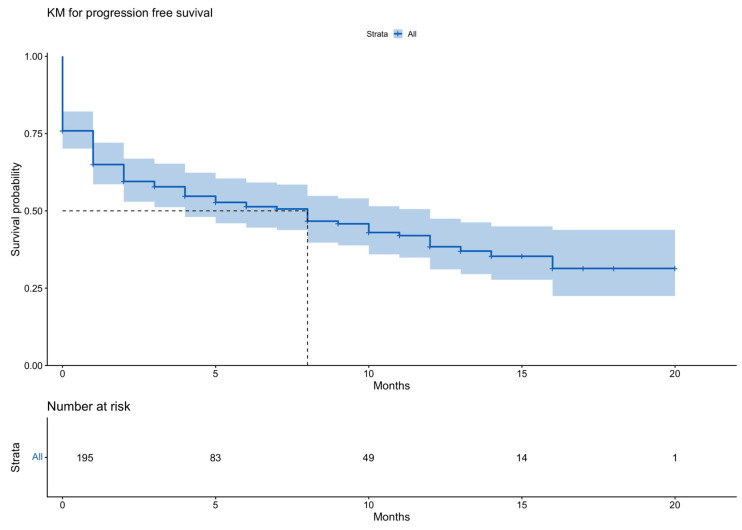
Kaplan–Meier curve for Progression Free Survival of teclistamab in the entire patient cohort.

**Figure 4:**
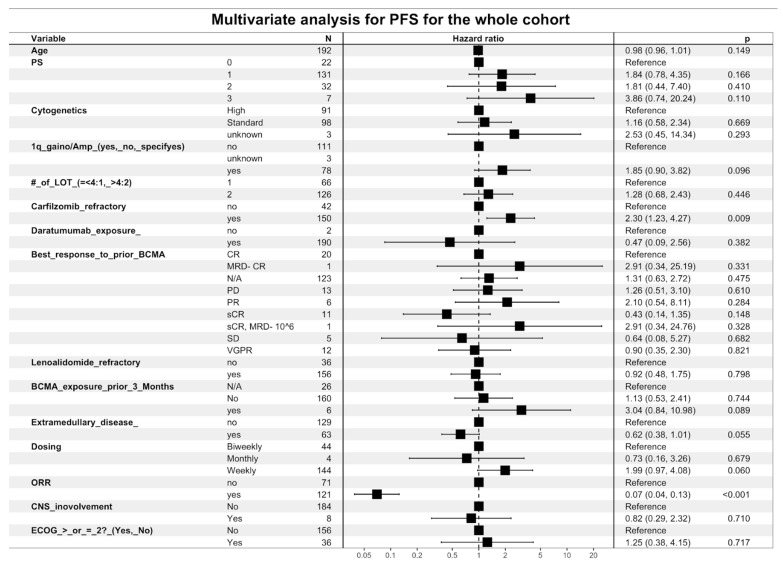
Multivariate analysis for Progression Free Survival of the entire patient cohort.

Notably, higher ORR correlated with longer PFS, not only in the univariate analysis of the entire patient cohort but also in the multivariate analysis. Figure 1 depicts overall survival (OS) outcomes, and Figure 2 represents the PFS outcomes with the use of teclistamab in both patient groups. Figure 3 demonstrates the PFS with the use of teclistamab in the entire patient cohort. Figure 4 shows the multivariate analysis of PFS for the entire patient cohort. Notably, there was a significant decline in the probability of OS and PFS upon treatment initiation, secondary to patient complications, progressive disease, patients being lost to follow-up or having missing data. Overall, 36% of the patients died: 50% of patients with RI and 34% of patients without RI. Early mortality largely reflects advanced disease burden, infection, or pre-treatment complications rather than treatment-related toxicity. Mortality within the first month of treatment occurred in 39% of patients: 50% of patients with RI and 37% of patients without RI. Supplementary Table S3 details the etiologies of mortality in patients with and without RI.

### 3.3. Toxicity Outcomes

Patients with RRMM and RI experienced toxicity profiles largely comparable to those without RI during teclistamab therapy. CRS occurred in nearly half of all patients (43.8% with RI vs. 52.5% without RI), though grade 1 CRS was less frequent among those with RI (35% vs. 47%, *p* = 0.5). The median onset of CRS was 4.5 days versus 3.8 days (*p* = 0.3), and the median duration was 1.1 days versus 1.6 days (*p* = 0.07 *) for patients with and without RI, respectively. ICANS occurred in 14.6% of RI patients and 10.6% of non-RI patients, with grade 3–4 events observed in 1% and 3%, respectively (*p* = 0.3).

Dexamethasone use during CRS management was significantly lower in patients with RI (12% vs. 28%, *p* = 0.048). Rates of recurrent CRS, step-up dose interruptions, and cumulative teclistamab exposure were similar across cohorts.

The incidence of infection did not differ significantly between groups (44% vs. 46%, *p* = 0.8). Severe infections occurred in 29% of RI patients and 17% of those without RI (*p* = 0.11). G-CSF support was required in 18% of RI patients and 33% of those without (*p* = 0.7), whereas IVIG was used more frequently among non-RI patients (57% vs. 38%, *p* = 0.052). PRBC transfusions were more common in patients with RI (50% vs. 24%, *p* = 0.003), likely reflecting baseline cytopenias. Platelet transfusion rates were similar (21% vs. 16%, *p* = 0.5).

At both 30 and 90 days, the incidence of grade 3/4 neutropenia, thrombocytopenia, and anemia was comparable between cohorts (all *p* > 0.1). IgG levels at 30 and 90 days showed no significant differences. Rates of hepatic, gastrointestinal, and neurologic toxicities were low and similar between groups. Hospitalization duration for the first teclistamab dose (median 7 days), frequency of ICU admission during step-up dosing (8.8% vs. 2.5%, *p* = 0.3), and frequency of dose delays (35% vs. 33%, *p* = 0.8) were comparable.

Overall, teclistamab was well tolerated in patients with RRMM and RI, with toxicity rates similar to those observed in patients without renal dysfunction. Supplementary Table S4 summarizes the toxicity outcomes of teclistamab in RRMM patients with and without RI.

## 4. Discussion

Previously published clinical studies on teclistamab use have largely excluded patients with RI, as seen in MajesTEC-1 [6]. Few case series with limited sample sizes have suggested the safety of teclistamab in patients with RI, particularly regarding CRS, ICANS, and infection rates [22,23]. The efficacy and safety of teclistamab have been reported in two systematic reviews and meta-analyses, both of which reported comparable ORR in subgroup and real-world analyses, with an overall manageable safety profile [24,25]. Notably, the five studies included by Qureshi et al. and the 34 studies by Li et al. in their systematic reviews and meta-analyses did not include RRMM with RI, as per their exclusion criteria, which included creatinine clearance or estimated GFR < 40 mL/min/1.73 m^2^.

A real-world, multi-institutional cohort of 303 European patients with RRMM, including 30 (9.9%) patients with severe RI, demonstrated superior OS and similar PFS to the results of MajesTEC-1, supporting the favorable safety and efficacy outcomes of teclistamab in patients with RI [26]. A similar real-world analysis in Czech patients demonstrated high efficacy comparable to the MajesTEC-1 trial results; however, it is unclear whether patients with RI were also considered [27].

Our study demonstrates the efficacy and safety outcomes of teclistamab in one of the largest published cohorts of patients with RRMM and RI. Patient demographics, including age, sex, race, myeloma type, and cytogenetics, were not statistically significant factors in determining the efficacy and safety outcomes between the RI and no RI groups. Our findings demonstrate that the efficacy and safety outcomes of teclistamab in RRMM with RI are comparable to those observed in patients with no RI.

When indexed to PFS outcomes in the univariate analysis, several statistically significant factors were identified, including performance status, 1q gain mutation, number of lines of therapy prior to teclistamab, carfilzomib refractory, and lenalidomide refractory patients. BCMA exposure and refractory patients, best response to prior BCMA, exposure to BCMA less than 3 or 6 months before teclistamab, and number of lines of treatment between the most recent BCMA and teclistamab were also significant factors. Furthermore, patients with penta-refractory disease, extramedullary disease, and ECOG performance status contributed significantly to PFS in this study. In the multivariate analysis, the presence of RI in RRMM was not statistically significant when indexed to PFS (HR 0.92, 95% CI, 0.66–1.43; *p* = 0.9). Our results did not demonstrate any significant difference in adverse events of CRS (*p* = 0.5) or ICANS (*p* = 0.3) of any grade in patients with or without RI treated with teclistamab.

Patients with and without RI appeared to have comparable overall and grade ≥ 3 leukopenia, neutropenia, anemia, and thrombocytopenia on days 30 and 90, respectively. TPO, GCSF, and IVIG use were comparable between the two groups. Although the anemia rates were similar between the two groups in our study, the frequency and use of pRBC transfusions were significantly higher in patients with RI than in those without RI (50% vs. 24%; *p* = 0.003). Overall, our study demonstrated that there was no significant difference in the grade of cytopenia, liver toxicity, GI toxicity, neuropathy, pancytopenia, hospitalization, or ICU admission rates between patients with RRMM with and without RI.

Dima et al. [28] reported efficacy and safety outcomes of RRMM in RI patients and severe RI patients (defined as CrCl < 30 mL/min or being on dialysis) when compared with patients with no RI. In the multivariate analysis, the presence of RI was not statistically significant when indexed to PFS (HR 1.2, 95% CI: 0.85–1.6, *p* = 0.31). The safety outcomes of CRS (*p* = 0.23), ICANS (*p* = 0.6), and mortality were comparable between groups. The incidence of grade ≥ 3 thrombocytopenia on day 30 (*p* = 0.002) was notably higher in patients with RI. However, the use of TPO, GCSF, and IVIG was comparable between the two groups in their study. The duration of treatment and reasons for discontinuation in both groups were not reported.

Pasvolsky et al. [27] reported the real-world efficacy and safety outcomes of the same patient groups upon completion of the two-year follow-up period. Although the results were compared between age groups (<75 and ≥75 years), the study included a total of 44 patients with severe RI. Although subgroup analysis for the RI group was not performed, the study demonstrated no significant differences in ORR, CRS, or ICANS between younger and older patient groups. Of the 385 patients, 20 aged ≥ 75 years and 118 aged < 75 years died. Of the patients aged ≥ 75 years, 14 died due to myeloma progression, five due to infection, and one due to other or unknown reasons. Among those aged < 75 years, 89 died due to myeloma progression, 12 due to infection, six due to other malignancies, and 11 due to other or unknown reasons.

Tan et al. [29] also reported a real-world evaluation of teclistamab for 210 RRMM patients, demonstrating ORR of 67% in 188 response-evaluable patients, 6-month PFS of 53%, 6-month OS of 73, CRS in 54% patients, infections in 56.2% patients, and 22% having grade ≥ 3 infections during the median follow-up of 5.3 months. Subgroup analysis of the 26 patients with CrCl ≤ 30 mL/min did not demonstrate any statistically significant differences in ORR, PFS, or OS compared with patients with CrCl > 30 mL/min. The reasons for treatment discontinuation were not reported in this study. In the MajesTEC-1 trial [6], 119 (72.1%) patients experienced CRS of all grades (1 [0.6%] of grade 3/4), and 24 (14.5%) experienced ICANS of all grades (1 [0.6%] of grade 3/4), all of which resolved with no treatment discontinuation. Additionally, infections occurred in 76.4% of patients, with grade 3/4 infections in 44.8% [5]. This toxicity profile, characterized by mostly grade 1 or 2 adverse events, was comparable to that observed in our study. Overall, these studies support the safety of teclistamab use in patients with RI, including those with severe RI and patients who require hemodialysis, which has also been recently shared by expert opinions [30].

In a multicenter study conducted by Snyder et al. [31], outcomes of a step-up dosage strategy followed by weekly administration of teclistamab in patients with RRMM for a fixed-duration versus continuous treatment were reported. The fixed-duration treatment group was defined as patients who achieved VGPR but had treatment discontinued due to deep response, toxicity, or preference. Continuous treatment was defined as patients achieving VGPR, with treatment continued until disease progression or intolerance to treatment. Their results demonstrated a higher CRR and shorter median time to best response in the fixed-duration group, with similar toxicity profiles in both groups, indicating the need to consider finite treatment in this patient group.

Our study also demonstrated that patients who responded to treatment had statistically significantly improved OS and PFS compared with those who did not respond to treatment, as demonstrated by the univariate and multivariate analyses of the entire patient cohort (Figure 3 and Figure 4).

Although quadruplet therapy has now become the standard for NDMM, and novel therapy options with BCMA-specific antibodies and CAR-T are being used for RRMM, on a global scale, financial burdens faced by patients and healthcare systems, along with a lack of approval from local regulatory agencies, have been cited as the primary barriers to accessing myeloma therapies [32]. This study highlights the real-world use of teclistamab, which may help guide the use of newer therapies when they are accessible globally.

This study had some limitations, including its retrospective and multi-institutional design, which may have introduced selection bias and unmeasured confounders, thereby affecting causal inference. The small sample size of patients with RI, especially those on dialysis, limits the statistical power for subgroup analyses and generalizability of the study. Variable follow-up durations may also underestimate long-term efficacy and late-onset toxicities. Treatment practice variations could influence toxicity rates, and the inability to differentiate renal function beyond the <40 mL/min threshold restricts outcome assessment. The impact of Teclistamab on potential renal recovery was not addressed in our study. Furthermore, data on treatment discontinuation, dose modifications, pharmacokinetics of teclistamab, and patient-reported outcomes were not obtained. The low frequency of high-grade CRS and ICANS events may have weakened statistical comparisons. Additionally, data pertaining to the erythropoietin trend during treatment with teclistamab in both patient groups were not measured in our study.

Despite these limitations, this study provides valuable insights into the safety and efficacy of teclistamab in patients with RRMM and RI, highlighting the need for further prospective evaluations and corroborating recent expert opinions on the use of teclistamab in patients with RRMM and RI.

## 5. Conclusions

In the multivariate analysis study, patients with RRMM with baseline RI did not show statistically significant factors affecting PFS. No notable differences were observed in the toxicity outcomes, including CRS, ICANS, and infections, between the RI and no RI groups. The significant findings included a higher number of pRBC transfusions and reduced dexamethasone administration in the RI group. Teclistamab demonstrated comparable efficacy and safety in patients with RRMM with RI and no RI, supporting their inclusion in future clinical trials with careful monitoring.

## Data Availability

The original contributions presented in this study are included in the article/Supplementary Material. Further inquiries can be directed to the corresponding authors.

## Supplementary Materials

The following supporting information can be downloaded at: https://www.mdpi.com/article/10.3390/cancers18050740/s1, Table S1: Patient Characteristics for All Patients with Relapsed/Refractory Multiple Myeloma with and without Renal Impairment Treated with Teclistamab; Table S2: Efficacy outcomes with Teclistamab in RRMM patients with renal impairment; Table S3: Etiology of Patient Mortality in Patients with and without RI; Table S4: Toxicity outcomes with Teclistamab in RRMM patients with and without renal impairment.
