# Supplementary material for: A Real-World Analysis of the Safety and Efficacy of Teclistamab for Patients with Relapsed/Refractory Multiple Myeloma and Baseline Renal Impairment—USMIRC Group
Source: Cancers (Basel). 2026 Feb 25;18(5):740. doi: 10.3390/cancers18050740 (PMC12984311; doi:10.3390/cancers18050740)
Supplement: Supplementary file 1 [file cancers-18-00740-s001.zip › cancers-4068577-supplementary.pdf]

Supplemental Table S1: Patient Characteristics for All Patients with Relapsed/Refractory Multiple Myeloma with and without Renal Impairment Treated with Teclistamab

**Characteristic**	**Overall (N = 195)**	**RRMM patients without RI (N = 161)**	**RRMM patients with RI (N = 34)**	**p-value**
**Age, Median (Range)**		69 (62-73)	71 (64-76)	0.093
**Gender, n (%)**				0.051
Female	81 (42%)	65 (40%)	16 (47%)	
Male	114 (58%)	96 (60%)	18 (11%)	
**Race**				0.5
African American	39 (20%)	30 (19%)	9 (26%)	
Asian	4 (2%)	4 (2%)	0 (0%)	
Hispanic	2 (1%)	1 (1%)	1 (3%)	
Caucasian	150 (77%)	126 (78%)	24 (71%)	
**Performance Status**				0.3
0	23 (12%)	22 (14%)	1 (3%)	
1	133 (68%)	108 (67%)	25 (74%)	
2	32 (16%)	26 (16%)	6 (18%)	
3	7 (4%)	5 (3%)	2 (6%)	
**Stage R-ISS**				0.6
1	39 (20%)	35 (22%)	4 (12%)	
2	69 (35%)	58 (36%)	11 (32%)	
3	49 (25%)	37 (23%)	12 (35%)	
Unknown	38 (19%)	31 (19%)	7 (21%)	
**Immunoglobulin type**				>0.9
IgA	39 (20%)	32 (20%)	7 (21%)	
IgG	-*	2 (24%)	-	
IgD	110 (56%)	88 (55%)	22 (65%)	
IgM	-	1 (1%)	-	
**Kappa/lambda**				>0.9
Kappa	107 (55%)	90 (56%)	17 (50%)	
Lambda	79 (41%)	62 (39%)	17 (50%)	
NA	9 (5%)	8 (5%)	1 (3%)	
**Cytogenetics**				0.4
High	93 (47%)	74 (46%)	19 (2%)	
Standard	99 (51%)	84 (52%)	15 (44%)	
unknown	3 (2%)	3 (2%)	0 (0%)	
**Del(17p)**				0.11
no	164 (84%)	135 (84%)	29 (85%)	
unknown	3 (2%)	3 (2%)	0 (0%)	
yes	28 (14%)	23 (14%)	5 (15%)	
**t(4;14)**				0.4

no	180 (9%)	149 (93%)	31 (91%)	
unknown	3 (2%)	3 (2%)	0 (0%)	
yes	12 (6%)	9 (6%)	3 (9%)	
**t(14;16)**				0.3
no	179 (92%)	146 (91%)	33 (97%)	
unknown	3 (2%)	3 (2%)	0 (0%)	
yes	13 (7%)	12 (7%)	1 (3%)	
**1q_gain/Amp**				>0.9
no	113 (58%)	95 (59%)	18 (53%)	
unknown	3 (2%)	3 (2%)	0 (0%)	
yes	79 (41%)	63 (39%)	16 (47%)	
**Prior ASCT**				0.6
no	70 (36%)	53 (33%)	17 (50%)	
yes	125 (64%)	108 (67%)	17 (50%)	
**Number of LOT prior to Tec**				>0.9
1	67 (34%)	57 (35%)	10 (29%)	
2	128 (66%)	104 (65%)	24 (71%)	
**Velcade exposure_**				0.3
no	4 (2%)	3 (2%)	1 (3%)	
yes	191 (98%)	158 (98%)	33 (97%)	
**Velcade refractory**				0.077
no	72 (37%)	63 (39%)	9 (26%)	
yes	122 (63%)	97 (60%)	25 (74%)	
**Carfilzomib exposure**				>0.9
No	26 (13%)	18 (11%)	8 (24%)	
Yes	169 (87%)	143 (89%)	26 (76%)	
**Carfilzomib refractory**				>0.9
No	42 (22%)	32 (20%)	10 (29%)	
Yes	153 (78%)	129 (80%)	24 (71%)	
**PI refractory**				0.08
no	31 (16%)	25 (16%)	6 (18%)	
yes	164 (84%)	136 (84%)	28 (82%)	
**Lenalidomide exposure**				0.12
no	5 (3%)	3 (2%)	2 (6%)	
yes	190 (98%)	158 (98%)	32 (94%)	
**Lenalidomide refractory**				0.8
no	36 (18%)	30 (19%)	6 (18%)	
yes	157 (81%)	130 (81%)	27 (79%)	
**Pomalidomide exposure**				0.2
no	30 (15%)	23 (14%)	7 (21%)	
yes	165 (85%)	138 (86%)	27 (79%)	
**Pomalidomide refractory**				0.7

no	41 (21%)	32 (20%)	9 (26%)	
yes	154 (79%)	129 (80%)	25 (74%)	
**IMiD refractory**				**0.049**
no	16 (8%)	13 (8%)	3 (9%)	
yes	179 (92%)	148 (92%)	31 (91%)	
**Daratumumab refractory**				0.12
no	10 (5%)	8 (5%)	2 (6%)	
yes	185 (95%)	153 (95%)	32 (94%)	
**BCMA exposure**				>0/9
no	124 (64%)	98 (61%)	26 (76%)	
yes	71 (36%)	63 (39%)	8 (24%)	
**BCMA_refractory_**				0.7
no	137 (70%)	110 (68%)	27 (79%)	
yes	58 (30%)	51 (32%)	7 (21%)	
**ADC (Blenrep)**				0.6
no	168 (86%)	141 (88%)	27 (79%)	
yes	27 (14%)	20 (12%)	7 (21%)	
**CAR-T**				0.2
Abecma	37 (19%)	34 (21%)	3 (9%)	
Carvykti	5 (3%)	5 (3%)	0 (0%)	
No	146 (75%)	115 (71%)	31 (91%)	
Other	7 (4%)	7 (4%)	0 (0%)	
**T-cell engagers_**				0.3
no	187 (96%)	154 (96%)	33 (97%)	
yes	8 (4%)	7 (4%)	1 (3%)	
**>=2_BCMA treatment**				>0.9
no	180 (92%)	149 (93%)	31 (91%)	
yes	15 (8%)	12 (7%)	3 (9%)	
**Best response to most recent prior BCMA**				0.3
CR	20 (10%)	18 (11%)	2 (6%)	
MRD-CR	1 (1%)	1 (1%)	0 (0%)	
N/A	125 (64%)	99 (61%)	26 (76%)	
PD	13 (7%)	11 (7%)	2 (6%)	
PR	6 (3%)	3 (2%)	3 (9%)	
sCR	11 (6%)	11 (7%)	0 (0%)	
MRD-sCR	1 (1%)	1 (1%)	0 (0%)	
SD	5 (3%)	5 (3%)	0 (0%)	
VGPR	13 (7%)	12 (7%)	1 (3%)	
**Exposure to BCMA <3 months prior to Tec**				0.4
N/A	27 (14%)	24 (15%)	3 (9%)	
no	162 (83%)	132 (82%)	30 (88%)	
yes	6 (3%)	5 (3%)	1 (3%)	

**Exposure to BCMA <6 months prior to Tec**				0.5
N/A	28 (14%)	24 (15%)	4 (12%)	
no	152 (78%)	124 (77%)	28 (82%)	
yes	15 (8%)	13 (8%)	2 (6%)	
**Number of LOT between most recent BCMA and Tec**				0.2
0	47 (24%)	40 (25%)	7 (21%)	
1	23 (12%)	19 (12%)	4 (12%)	
2	-	8 (5%)	-	
3	-	4 (2%)	-	
3+	-	5 (3%)	-	
N/A	106 (54%)	85 (53%)	21 (62%)	
**Double refractory**				**0.041**
no	31 (16%)	24 (15%)	7 (21%)	
yes	164 (84%)	137 (85%)	27 (79%)	
**Triple RRMM**				0.077
no	40 (21%)	31 (19%)	9 (26%)	
yes	155 (79%)	130 (81%)	25 (74%)	
**Penta-exposed**				0.5
no	60 (31%)	47 (29%)	13 (38%)	
yes	135 (69%)	114 (71%)	21 (62%)	
**Penta-refractory**				0.8
no	102 (52%)	82 (51%)	20 (59%)	
yes	93 (48%)	79 (49%)	14 (41%)	
**Extramedullary disease**				0.11
no	131 (67%)	102 (63%)	29 (85%)	
yes	64 (33%)	59 (37%)	5 (15%)	
**Plasma cell leukemia**				0.3
no	188 (96%)	155 (96%)	33 (97%)	
yes	7 (4%)	6 (4%)	1 (3%)	
**Amyloidosis**				0.3
no	193 (99%)	160 (99%)	33 (97%)	
yes	2 (1%)	1 (1%)	1 (3%)	
**Step up dosing schedule**				0.8
1,3,5	117 (60%)	90 (56%)	27 (79%)	
1,4,7	78 (40%)	71 (44%)	7 (21%)	
**ICU admission**				0.4
no	188 (96%)	157 (98%)	31 (91%)	
yes	7 (4%)	4 (2%)	3 (9%)	
**Dosing**				**0.022**
Biweekly	44 (23%)	38 (24%)	6 (18%)	
Monthly	4 (2%)	3 (2%)	1 (3%)	
Weekly	147 (75%)	120 (75%)	27 (79%)	

**ORR**				**<0.001**
no	72 (37%)	57 (35%)	15 (44%)	
yes	122 (63%)	103 (64%)	19 (56%)	
**ECOG ≥2**				0.2
no	159 (82%)	135 (84%)	24 (71%)	
yes	36 (18%)	26 (16%)	10 (29%)	

Abbreviations: *GFR:* Glomerular Filtration Rate; *IMiD:* Immunomodulatory Drugs; *ISS-R:* Revised International Staging System; *ASCT:* Autologous Stem Cell Transplant; *LOT*: Lines of Therapy; *Tec*: Teclistamab; *PI*: Proteasome Inhibitor; *BCMA*: B-cell Maturation Agent; *ADC*: Antibody-Drug Conjugate; *CR*: Complete Response; *MRD*: Minimal Residual Disease; *PD*: Progressive Disease; *PR*: Partial Response; *sCR*: Serum Complete Response; *SD*: Stable Disease; *VGPR*: Very Good Partial Response; *RRMM*: Relapsed Refractory Multiple Myeloma; *ORR*: Overall Response Rate; *ECOG*: Eastern Cooperative Oncology Group

*Data label "-" refers to the data not being available due to the patient being lost to follow-up, patient death, or myeloma not measurable at baseline.

Supplemental Table S2: Efficacy outcomes with Teclistamab in RRMM patients with renal impairment

Patient characteristic	PFS Univariate Analysis			
	RRMM patients with RI (N = 34)			
	**N**	**HR**	**95% CI**	**P-value**
**Gender**				0.051
Female	16	-	-	
Male	18	2.53	0.96-6.68	
**Race**				0.5
African American	9	-	-	
Asian				
Hispanic	1	0	0.00-Inf	
White	24	0.84	0.30-2.34	
**Performance Status**				0.3
0	1	-	-	
1	25	0.2	0.02-1.60	
2	6	0.46	0.05-4.15	
3	2	0.53	0.05-5.99	
**Stage R-ISS**				0.6
1	4	-	-	
2	11	2.39	0.29-19.6	
3	12	3	0.37-24.1	
Unknown	7	1.65	0.17-16.0	
**Immunoglobulin type**				>0.9
IgA	7	-	-	
IgG				
IgD	22	0.77	0.25-2.41	
IgM				
**Kappa/lambda**				>0.9
Kappa	17	-	-	
Lambda	17	1.02	0.42-2.52	
NA				
**Cytogenetics**				0.4
High	19	-	-	
Standard	15	0.68	0.27-1.73	
unknown				
**Del(17p)**				0.11
no	29	-	-	
unknown				
yes	5	2.77	0.88-8.66	
**t(4;14)**				0.4

no	31	-	-	
unknown				
yes	3	1.68	0.49-5.77	
**t(14;16)**				0.3
no	33	-	-	
unknown				
yes	1	3.83	0.50-29.5	
**1q_gain/Amp**				>0.9
no	18	-	-	
unknown				
yes	16	1.01	0.41-2.49	
**Prior ASCT**				0.6
no	17	-	-	
yes	17	0.79	0.32-1.94	
**Number of LOT prior to Tec**	34	1	0.84-1.19	>0.9
1	10	-	-	
2	24	0.74	0.28-1.96	
**Velcade exposure_**				0.3
no	1	-	-	
yes	33		0.00-Inf	
**Velcade refractory**				0.077
no	9	-	-	
yes	25	3.18	0.73-13.9	
**Carfilzomib exposure**				>0.9
No	8	-	-	
Yes	26	1	0.33-3.03	
**Carfilzomib refractory**				>0.9
No	10	-	-	
Yes	24	0.98	0.35-2.73	
**PI refractory**				0.08
no	6	-	-	
yes	28	4.25	0.56-32.0	
**Lenalidomide exposure**				0.12
no	2	-	-	
yes	32	0.24	0.05-1.10	
**Lenalidomide refractory**				0.8
no	6	-	-	
yes	27	1.22	0.35-4.20	
**Pomalidomide exposure**				0.2
no	7	-	-	
yes	27	0.51	0.18-1.46	
**Pomalidomide refractory**				0.7
no	9	-	-	
yes	25	0.78	0.27-2.25	

**IMiD refractory**				**0.049**
no	3	-	-	
yes	31		0.00-Inf	
**Daratumumab refractory**				0.12
no	2	-	-	
yes	32	0.24	0.05-1.10	
**BCMA exposure**				>0/9
no	26	-	-	
yes	8	0.99	0.35-2.76	
**BCMA_refractory_**				0.7
no	27	-	-	
yes	7	0.79	0.26-2.41	
**ADC (Blenrep)**				0.6
no	27	-	-	
yes	7	1.32	0.48-3.68	
**CAR-T**				0.2
Abecma	3	-	-	
Carvykti				
No	31	3.31	0.43-25.4	
Other				
**T-cell engagers_**				0.3
no	33	-	-	
yes	1	3.83	0.50-29.5	
**>=2_BCMA treatment**				>0.9
no	31	-	-	
yes	3	0.93	0.21-4.08	
**Best response to most recent prior BCMA**				0.3
CR	2	-	-	
MRD-CR				
N/A	26	0.9	0.20-4.00	
PD	2	3.38	0.45-25.7	
PR	3	0.46	0.04-5.11	
sCR				
MRD-sCR				
SD				
VGPR	1	0	0.00-Inf	
**Exposure to BCMA <3 months prior to Tec**				0.4
N/A	3	-	-	
no	30	1.95	0.26-14.6	
yes	1	7.1	0.43-117	
**Exposure to BCMA <6 months prior to Tec**				0.5
N/A	4	-	-	

no	28	2.82	0.82-21.2	
yes	2	2.28	0.14-36.8	
**Number of LOT between most recent BCMA and Tec**				0.2
0	7	-	-	
1	4	0.23	0.03-1.97	
2				
3				
3+				
N/A	21	0.72	0.25-2.08	
**Double refractory**				**0.041**
no	7	-	-	
yes	27	5.16	0.68-38.9	
**Triple RRMM**				0.077
no	9	-	-	
yes	25	3.18	0.73-13.9	
**Penta-exposed**				0.5
no	13	-	-	
yes	21	0.71	0.28-1.77	
**Penta-refractory**				0.8
no	20	-	-	
yes	14	0.89	0.36-2.24	
**Extramedullary disease**				0.11
no	29	-	-	
yes	5	2.77	0.88-8.66	
**Plasma cell leukemia**				0.3
no	33	-	-	
yes	1	3.83	0.50-29.5	
**Amyloidosis**				0.3
no	33	-	-	
yes	1	3.83	0.50-29.5	
**Step up dosing schedule**				0.8
1,3,5	27	-	-	
1,4,7	7	1.13	0.40-3.19	
**ICU admission**				0.4
no	31	-	-	
yes	3	1.86	0.54-6.43	
**Dosing**				**0.022**
Biweekly	6	-	-	
Monthly	1	0	0.00-Inf	
Weekly	27	4.92	1.04-23.4	
**ORR**				**<0.001**
no	15	-	-	
yes	19	0	0.00-Inf	
**ECOG ≥2**				0.2

no	24	-	-	
yes	10	1.81	0.71-4.63	

Abbreviations: *GFR:* Glomerular Filtration Rate; *PFS:* Progression-Free Survival; *HR:* Hazard Ratio; *CI:* Confidence Interval; *IMiD:* Immunomodulatory Drugs; *ISS-R:* Revised International Staging System; *ASCT:* Autologous Stem Cell Transplant; *LOT*: Lines of Therapy; *Tec*: Teclistamab; *PI*: Proteasome Inhibitor; *BCMA*: B-cell Maturation Agent; *ADC*: Antibody-Drug Conjugate; *CR*: Complete Response; *MRD*: Minimal Residual Disease; *PD*: Progressive Disease; *PR*: Partial Response; *sCR*: Serum Complete Response; *SD*: Stable Disease; *VGPR*: Very Good Partial Response; *RRMM*: Relapsed Refractory Multiple Myeloma; *ORR*: Overall Response Rate; *ECOG*: Eastern Cooperative Oncology Group

*Data label “-“ refers to the data not being available due to the patient being lost to follow-up, patient death, or myeloma not measurable at baseline.

Supplemental Table S3: Etiology of Patient Mortality in Patients with and without RI

**Etiology of Patient Mortality**	**RRMM patients without RI (N=161)**	**RRMM patients with RI (N = 34)**
PD	36 (22%)	13 (38%)
Neurotoxicity	2 (1%)	1 (3%)
Respiratory failure/PNA	7 (4%)	N/A
Infection without sepsis	2 (1%)	N/A
COVID	1 (1%)	N/A
Sepsis/septic shock	2 (1%)	1 (3%)
Subarachnoid hemorrhage	N/A	1 (3%)
Unknown	2 (1%)	N/A
Bilateral PE/atrial thrombus	1 (1%)	N/A
Pneumatosis intestinalis	1 (1%)	N/A
CMV viremia	1 (1%)	1 (3%)
Multiorgan failure	N/A	1 (3%)
Cirrhosis	1 (1%)	N/A
Vfib	N/A	1 (3%)
AML	1 (1%)	N/A

Abbreviations: *PD: Progressive Disease; PNA: Pneumonia, COVID; Coronavirus, PE: Pulmonary Embolism; CMV: Cytomegalovirus; Vfib: Ventricular Fibrillation; AML: Acute Myeloid Leukemia; N/A: Not Applicable*

Supplemental Table S4: Toxicity outcomes with Teclistamab in RRMM patients with and without renal impairment

**Patient toxicity based on renal function**	**RRMM patients without RI (N=161)**	**RRMM patients with RI (N = 34)**	**P-value**
**Liver toxicity (n=195)**			0.6
Grade 0	137 (85%)	28 (82%)	
Grade 1	18 (11%)	6 (18%)	
Grade 2	4 (2.5%)	0 (0%)	
Grade 3	1 (0.6%)	0 (0%)	
None	1 (0.6%)	0 (0%)	
**GI toxicity (n=195)**	6 (3.7%)	3 (8.8%)	0.2
**Neutropenic fever (n=195)**	5 (3.1%)	1 (2.9%)	>0.9
**CRS (n=195)**			0.5
Grade 0	74 (46%)	19 (56%)	
Grade 1	75 (47%)	12 (35%)	
Grade 2	7 (4.3%)	2 (5.9%)	
Grade 3	2 (1.2%)	1 (2.9%)	
None	3 (1.9%)	0 (0%)	
**ICANS (n=195)**			0.3
Grade 0	144 (89%)	29 (85%)	
Grade 1	8 (5.0%)	3 (8.8%)	
Grade 2	6 (3.7%)	1 (2.9%)	
Grade 3	3 (1.9%)	0 (0%)	
Grade 4	0 (0%)	1 (2.9%)	
**Tocilizumab treatment (n=195)**	44 (27%)	12 (35%)	0.4
**Number of tocilizumab treatments (n=195)**	0.34 (0.00, 4.00)	0.41 (0.00, 2.00)	0.4
**Recurrent CRS after Tocilizumab doses**	8 (5.0%)	1 (2.9%)	>0.9
**Dexamethasone treatment (n=195)**	45 (28%)	4 (12%)	**0.048**
**Neuropathy (n=195)**	3 (1.9%)	2 (5.9%)	0.2
**Leukopenia at day 30 (n=175)**			0.3
Grade 0	77 (52%)	18 (67%)	
Grade 1	35 (24%)	3 (11%)	
Grade 2	26 (18%)	3 (11%)	
Grade 3	7 (4.7%)	3 (11%)	
Grade 4	3 (2.0%)	0 (0%)	
Unknown	13 (7.4%)	7	
**Anemia at day 30 (n=175)**			0.5
Grade 0	60 (41%)	7 (26%)	
Grade 1	38 (26%)	10 (37%)	
Grade 2	35 (24%)	8 (30%)	
Grade 3	14 (9.5%)	2 (7.4%)	

Grade 4	1 (0.7%)	0 (0%)	
Unknown	13 (7.4%)	7 (4%)	
**Neutropenia at day 30 (n=175)**			0.6
Grade 0	101 (68%)	21 (78%)	
Grade 1	17 (11%)	3 (11%)	
Grade 2	13 (8.8%)	0 (0%)	
Grade 3	12 (8.1%)	2 (7.4%)	
Grade 4	5 (3.4%)	1 (3.7%)	
Unknown	13 (7.4%)	7 (4%)	
**Thrombocytopenia at day 30 (n=175)**			0.3
Grade 0	75 (51%)	12 (44%)	
Grade 1	33 (22%)	9 (33%)	
Grade 2	21 (14%)	2 (7.4%)	
Grade 3	10 (6.8%)	4 (15%)	
Grade 4	9 (6.1%)	0 (0%)	
Unknown	13 (7.4%)	7 (4%)	
**Leukopenia at day 90 (n=125)**			0.2
Grade 0	74 (70%)	15 (79%)	
Grade 1	21 (20%)	1 (5.3%)	
Grade 2	7 (6.6%)	1 (5.3%)	
Grade 3	3 (2.8%)	2 (11%)	
Grade 4	1 (0.9%)	0 (0%)	
Unknown	55 (44%)	15 (8.6%)	
**Anemia at day 90 (n=125)**			0.2
Grade 0	64 (60%)	8 (42%)	
Grade 1	26 (25%)	6 (32%)	
Grade 2	13 (12%)	3 (16%)	
Grade 3	3 (2.8%)	2 (11%)	
Unknown	55 (44%)	15 (8.6%)	
**Neutropenia at day 90 (n=125)**			0.4
Grade 0	83 (78%)	15 (79%)	
Grade 1	8 (7.5%)	0 (0%)	
Grade 2	6 (5.7%)	2 (11%)	
Grade 3	4 (3.8%)	2 (11%)	
Grade 4	5 (4.7%)	0 (0%)	
Unknown	55 (44%)	15 (8.6%)	
**Thrombocytopenia at day 90 (n=125)**			0.2
Grade 0	67 (63%)	14 (74%)	
Grade 1	31 (29%)	2 (11%)	
Grade 2	5 (4.7%)	2 (11%)	

Grade 3	2 (1.9%)	1 (5.3%)	
Grade 4	1 (0.9%)	0 (0%)	
Unknown	55 (44%)	15 (8.6%)	
**Use of GCSF (n=195)**	33 (20%)	6 (18%)	0.7
**Use of TPO agonists (n=195)**	4 (2.5%)	0 (0%)	>0.9
**Use of IVIG (n=195)**	91 (57%)	13 (38%)	0.052
**PRBC transfusion (n=195)**	39 (24%)	17 (50%)	**0.003**
**Platelet transfusion (n=195)**	25 (16%)	7 (21%)	0.5
**Infection (n=195)**	74 (46%)	15 (44%)	0.8
**Severe infection (n=195)**	28 (17%)	10 (29%)	0.11
**Hospitalization at first dose**	159 (99%)	33 (97%)	0.4
**Hospitalization at subsequent doses (n=195)**	42 (26%)	11 (32%)	0.6

Abbreviations: *GFR*: Glomerular Filtration Rate; *GI*: Gastrointestinal; *CRS*: Cytokine Release Syndrome; *ICANS:* Immune effector Cell-Associated Neurotoxicity Syndrome; *GCSF:* Growth Colony Stimulating Factor; *TPO:* Thrombopoietin; *IVIG:* Intravenous Immunoglobulin; *PRBC:* Packed Red Blood Cell

*Note: Data that was not available in the table was due to the patient being lost to follow-up, patient death, or myeloma not measurable at baseline.
